# A clinical study of genetic testing to guide the dosing of warfarin after heart valve replacement

**DOI:** 10.1186/s12872-022-02620-x

**Published:** 2022-04-19

**Authors:** Fan Zhang, Congcong Zhang, Chengxiong Gu, Yang Yu, Jingxing Li

**Affiliations:** Department of Cardiac Surgery, Beijing Anzhen Hospital, Capital Medical University, Anzhen Road #2, Beijing, 100029 China

## Abstract

**Objective:**

To explore the role of genetic testing of *VKORC1* and *CYP2C9* in determining the dosage of warfarin after aortic valve replacement.

**Methods:**

A total of 172 patients receiving warfarin after aortic valve replacement were divided into a control group (n = 86) and an experimental (n = 86) group based on acceptance of genetic testing. In the experimental group, three loci of *VKORC1* and *CYP2C9* were tested by polymerase chain reaction-restriction fragment length polymorphism technique, and the initial dose of warfarin was determined based on the genetic testing results and warfarin oral-dose table recommended by U.S. Food and Drug Administration (FDA). In the control group, warfarin (3 mg per night) was used as the initial dose. The international normalized ratio (INR) of each patient was continuously monitored after medication. The percentages of patients meeting the target INR in the two groups at specific time points and at 3-month follow-up after discharge from the hospital were monitored, and the incidence of various adverse events was compared between the groups.

**Results:**

Based on the results of genetic testing, 68 patients received 3–4 mg/d (79.1%), 10 patients received 0.5–2 mg/d (11.6%), and eight patients received 5–7 mg/d (9.3%) as the initial dosages of warfarin in the experimental group. The percentages of the patients meeting the target INR on the third and sixth day of postoperative medication were 45.3% and 73.3%, respectively, in the experimental group, and 29.8% and 58.3%, respectively, in the control group. The INR critical values during hospitalization occurred in 2.3% in the experimental group and in 7.1% in the control group, while the percentage of the patients meeting the target INR after 3 months was 86.1% in the experimental group and 83.1% in the control group.

**Conclusion:**

Genetic testing may guide the selection of the initial dose of warfarin after heart valve replacement to rapidly achieve a stable dose.

## Introduction

As a coumarin, a vitamin-K antagonist, warfarin is a commonly used anticoagulant drug in clinical practice, especially after mechanical valve replacement. Given that the body responds to a prosthetic valve as to a foreign substance, arteriovenous and cardiac thrombotic events may occur after mechanical valve replacement. To prevent such events, preoperative application of warfarin before heart valve replacement and life-long warfarin use after the replacement surgery are generally required. However, due to the differences between individuals and the narrow therapeutic window, precise dosage is challenging. Excessive dose of warfarin increases the risk of bleeding, while insufficient dose results in ineffective prevention of thrombosis. Hence, to optimize the effectiveness of warfarin and reduce the risk of adverse events, clinical application of warfarin for anticoagulant treatment requires careful monitoring of the international normalization ratio (INR) of prothrombin time (PT-INR) and continuous adjustment of the dosage of warfarin until the INR reaches the target value [1].

However, the dosage of warfarin is affected by various factors, such as gender, age, body mass index, diet, and concomitant medications [2]. In addition, genetic factors also play an important role in the process of warfarin metabolism, thereby affecting the variability of warfarin dosage. Genetic variations in the cytochrome P450 family 2, subfamily C, polypeptide 9 (*CYP2C9*) and vitamin K epoxide reductase complex subunit 1 (*VKORC1*) genes are significantly associated with the dose adjustment of warfarin because the products of these two genes play important roles in the pharmacokinetics and pharmacodynamics of warfarin [3]. It has been shown that genetic testing of three loci of these two genes, including VKORC1 (c.-1639G/A), CYP2C9*2 (430C/T), and CYP2C9*3 (1075A/C), can help to determine the recommended dosage of warfarin in clinical practice [4, 5]. Genotype-guided warfarin dosing may lead to safer anticoagulation and potentially improve outcomes in the implantation of continuous-flow left ventricular assist devices [6] and atrial fibrillation [7]. However, it is still unknown whether the genetic testing can guide the use of warfarin after aortic valve replacement and improve the outcome.

In this study, the percentage of patients meeting the target INR and the incidence of critical INR values after aortic valve replacement were analyzed in patients with or without genetic tests for *VKORC1* and *CYP2C9*. This study will provide an evidence for the use of genetic testing to guide warfarin dosage after aortic valve replacement.

## Material and methods

### Clinical data

Since 2019, our team has used genetic testing to determine the initial dosage of warfarin in patients after heart valve replacement. This study included 172 patients who underwent mechanical aortic valve replacement in the Center for Cardiac Surgery, Beijing Anzhen Hospital, Beijing, China from 2019 to 2020. Patients who had hepatic dysfunction, cirrhosis, renal insufficiency, coagulation dysfunction, thyroid disease, or cancer, or who received drug therapy that interacted with warfarin were excluded from this study. Cardiac examination was performed using color Doppler ultrasonography, and the patients signed written informed consent before participating in the study. Preoperative baseline clinical data of the patients were recorded and shown in Table 1. This study was reviewed and approved by the Ethics Committee of Beijing Anzhen Hospital (Num: 2019084X).

**Table 1:** Clinical information of the patients included in the study

Item	Control group (n = 86)	Experimental group (n = 86)	P value
Male, n (%)	50 (58.14)	55 (63.95)	0.532
Age (year)	52 ± 12	53 ± 12	0.758
Nationality-Han (%)	100	100	
Residence			
Urban	57	60	0.735
Rural	29	26	
Body mass index (kg/m^2^)	24.7 ± 2.5	25.3 ± 3.9	0.64
Smoking history	34 (39.53)	30 (34.88)	0.636
Alcohol consumption history	29 (33.72)	23 (26.74)	0.407
Aortic stenosis	20 (23.26)	16 (18.60)	0.471
Aortic insufficiency	43 (50.00)	51 (59.30)	
Aortic stenosis combined with aortic insufficiency	23 (26.74)	19 (22.09)	

Among the 172 patients, 86 patients were subjected to genetic testing for warfarin sensitivity after obtaining their informed consent for the testing and before the aortic valve replacement. The three tested loci were VKORC1 (c-1639G/A), CYP2C9*2 (430C/T), and CYP2C9*3 (1075A/C), and the specific variants were determined. This group of patients was classified as the experimental group, and it included 53 males and 33 females. The dosage of warfarin was guided by the test results. The corresponding dose of warfarin was given to the patient as the initial dosage when the patient resumed enteral nutrition (including oral and gastric tube feeding) after the replacement surgery. In addition, daily INR testing was conducted, and the daily dosage of warfarin was adjusted with the target INR value between 1.5 and 2.5 until the patient was discharged.

The remaining 86 patients who did not agree to the genetic testing were classified as the control group, including 50 males and 36 females. The patients in the control group were given 3 mg/d warfarin as the initial dose once they resumed enteral nutrition after the replacement surgery. Similar to the experimental group, the daily INR testing was conducted, and the daily dosage of warfarin was adjusted with the target INR value between 1.5 and 2.5 until the patient was discharged. The number and percentage of the patients reaching the target INR on the third day and the sixth day of postoperative medication in the two groups of patients were recorded. Moreover, INR > 4 was considered a critical value, and the number of patients with critical INR values was recorded in each group.

### DNA extraction, VKORC1 and CYP2C9 genotyping

5 mL venous blood was taken from patients and genomic DNA was extracted from the blood and standardized to approximately 100 ng/µl for polymerase chain reaction (PCR). Exons of CYP2C9 and promoter region of VKORC1 were amplified and sequenced using corresponding primers. The amplified products were purified using the gel purification system and sequenced using the ABI Prism Big Dye Terminator Cycler Sequencing Kit (Applied Biosystems) on the ABI 3730xl DNA Analyzer. All the sequences data were verified by at least two individuals.

### Postoperative follow-up

All of the patients were required to continue with the INR testing after being discharged, and the daily dosage of warfarin was adjusted to maintain the target INR value (between 1.5 and 2.5). The INR testing frequency was once every 5 to 7 days during the first month after discharge, and it was changed to once every 10 to 13 days from the second month after discharge provided that the testing index was stable. The frequency of INR testing was changed to once per month if the testing index was stable 3 months after discharge. Routine follow-up examinations, including color Doppler ultrasonography, electrocardiogram, orthographic chest radiography, and INR testing were performed at least 3 months after the replacement surgery. The INR results and the dosage of warfarin taken by each patient during 3 months of postoperative follow-up were recorded.

### Statistical analysis

The recommended dosages of warfarin guided by the warfarin sensitivity testing in the patients of the experimental group, the number of patients (among all patients) who reached the target INR on the third and the sixth days of postoperative medication, the frequency of critical INR values occurring during the perioperative period, and the data of warfarin dosage and INR during 3 months of postoperative follow-up were summarized. SPSS 21.0 statistical software was used for the statistical analysis. Continuous variables were presented as mean ± standard deviation, and the count data were presented as percentage. Continuous variables were compared between multiple groups using one-way ANOVA. Dichotomous variables were compared using the chi-square test. Comparisons between two groups were performed using paired *t* test. P values less than 0.05 were considered as statistically significant.

## Results

### Results of preoperative genetic testing

As shown in Table 1, there were 86 patients in each of the experimental and control groups. In this study, their baseline data of the patients did not show significant differences (p > 0.05), which indicated that the data of the two groups are comparable.

Preoperative blood samples were collected from 86 patients in the experimental group for DNA extraction, followed by warfarin sensitivity testing of three genetic loci, including VKORC1, CYP2C9*2, and CYP2C9*3. For the c.-1639 locus of *VKORC1*, AA was found in 78 patients (90.7%), GA was found in 7 patients (8.1%), and only 1 patient had GG (1.2%). For *CYP2C9*, there were 76 patients with the wild-type genotype (CYP2C9*1/*1) (88.4%), 10 patients carrying CYP2C9*1/*3 (11.6%), and no other genotypes were detected (Table 2).

**Table 2:** Distribution of genotypes of the patients

VKORC1 c.-1639G/A^a^	CYP2C9^b^					
	*1/*1	*1/*2	*1/*3	*2/*2	*2/*3	*3/*3
GG	1	0	0	0	0	0
GA	7	0	0	0	0	0
AA	68	0	10	0	0	0

^a^G at the nucleotide position -1639 of the gene promoter is changed to A

^b^The CYP2C9*1 allele (i.e., the wild-type allele) is associated with normal enzyme activity; in the CYP2C9*2 allele, C at the nucleotide position 403 of the gene is changed to T; in the CYP2C9*3 allele, A at the nucleotide position 1075 of the gene is changed to C

### Determination of warfarin dosage based on the genotypes

Based on the combined results of several genes and the FDA-recommended table for the oral dosage of warfarin, in the experimental group, 68 patients were recommended to receive 3–4 mg/d (79.1%) as the initial dosage of warfarin, while 10 and 8 patients were recommended to receive 0.5–2 mg/d (11.6%) and 5–7 mg/d (9.3%), respectively, as the initial dosage of warfarin (Table 3). Considering that each warfarin tablet contains 3 mg, we rounded the initial dosage to facilitate medication. Specifically, 3 mg/d warfarin (1 tablet) was the initial dosage for the patients of the 3–4 mg/d group; 1.5 mg/d warfarin (0.5 tablet) was the initial dosage for the patients of the 0.5–2 mg/d group; and 6 mg/d warfarin (2 tablets) was the initial dosage for the patients of the 5–7 mg/d group.

**Table 3:** FDA recommended daily warfarin dosage (mg/d) based on *VKORC1* and *CYP2C9* genotypes

VKORC1 c.-1639G/A	CYP2C9 *1/*1	CYP2C9 *1/*2	CYP2C9 *1/*3	CYP2C9 *2/*2	CYP2C9 *2/*3	CYP2C9 *3/*3
GG	5–7	5–7	3–4	3–4	3–4	0.5–2
GA	5–7	3–4	3–4	3–4	0.5–2	0.5–2
AA	3–4	3–4	0.5–2	0.5–2	0.5–2	0.5–2

### Percentage of the patients meeting the target INR and incidence of critical INR values occurring perioperatively

**Fig. 1:**
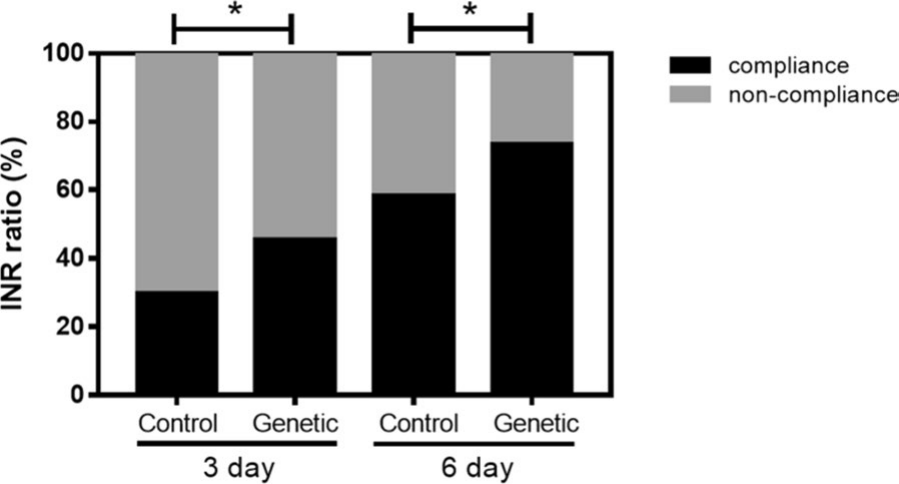
Percentage of the patients meeting the target INR on the third and sixth days of postoperative medication

Two patients in the control group died intraoperatively, so a total of 84 patients in the control group were included in the follow-up study. On the third day of postoperative medication, there were 23 patients in the control group (29.8%) with the INR values within the target range (1.5–2.5), and there were 39 patients in the experimental group (45.3%) with INR values within the target range between 1.5 and 2.5 (p < 0.05). On the sixth day of postoperative medication, there were 49 patients in the control group (58.3%) within the target INR range, and 63 patients in the experimental group (73.3%) within the target INR range (p < 0.05) (Fig. 1). During the hospitalization, 6 patients in the control group (7.1%) and 2 patients in the experimental group (2.3%) developed critical INR values (p < 0.05), the incidence of critical INR values was significantly lower (by 3.09-fold) in the experimental group.

### Follow-up results at 3 months postoperatively

At 3 months after operation, the INR values and warfarin doses were collected from 144 patients, including 79 patients from the experimental group and 65 patients from the control group. A total of 16 patients were lost to follow-up (9.4% lost rate), which included 11 in the control group and 5 in the experimental group. 10 patients were died, which included 6 in the control group and 4 in the experimental group. The death causes of 6 patients of control group included: 1 case of cerebral infarction (in-hospital), 1 case of severe pneumonia (in-hospital), 1 case of gastrointestinal hemorrhage (after discharge), and 3 cases of cerebral hemorrhage (2 cases after discharge, 1 case in hospital). The death causes of 4 patients of experimental group included: 1 case of cerebral infarction (in-hospital), 1 case of severe pneumonia (in-hospital), 1 case of cerebral hemorrhage (after discharge), and 1 case of sudden death after discharge, and the cause of death was unknown (after discharge).

**Fig. 2:**
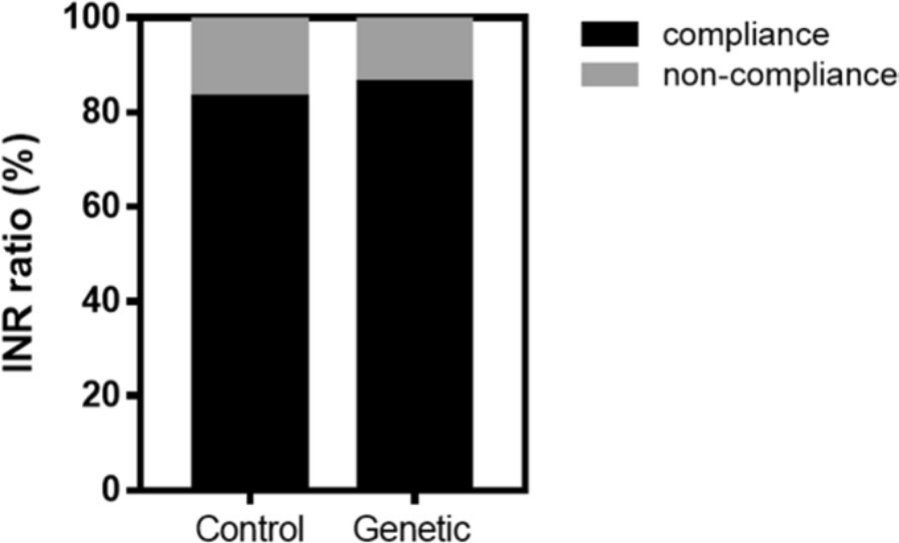
Percentage of the patients meeting the target INR on the 30th day of postoperative medication

Among the patients with reported results in the follow-up analysis, 54 patients (83.1%) in the control group and 68 patients (86.1%) in the experimental group achieved the target INR range (Fig. 2). These results indicated no significant difference between the two groups (p = 0.65).

## Discussion

Reaching a stable and effective warfarin dosage as soon as possible could reduce the incidence of postoperative adverse cardiovascular events and improve the prognosis of valve replacement surgery. In this study, we using the genetic testing result for *VKORC1* and *CYP2C9* to determine the recommended dosage of warfarin after AVR.

The most commonly used strategy is to perform genetic testing on three loci of two genes, including VKORC1(c-1639G/A), CYP2C9*2(430C/T), and CYP2C9*3(1075A/C) [8]. The data shown that, among the 86 patients with genetic testing, 90.7% patients were AA allele for the c.-1639 locus of *VKORC1*, 11.6% patients were AC allele for 1075 locus of *CYP2C9* and no CT or TT allele for 403 locus of *CYP2C9*. This result is consistent with the previous study that the VKORC1 (c.-1639AA) genotype is widely distributed in Chinese populations (83.70%) and there is almost no CYP2C9*2 allele in Chinese populations [9]. Another study also shown that the VKORC1 (c.-1639AA) genotype was found in 89.10% Japanese [10]. And the CYP2C9 *2 allele was the most prevalent genotype in the Iranian population [11]. These data indicated that, for the difference in ethnic, we should adjust the warfarin dose more suitable for Chinese people according to genotype.

According to genotype-oriented algorithm provided by The International Warfarin Pharmacogenetics Consortium (IWPC) [12], 10 patients (11.6%) received a lower dosage, and 8 patients (9.3%) received a higher dosage as the initial dosages of warfarin in the experimental group. Under this initial dosage, more patients achieved the target INR values at 3 days and 6 days after operation in experiment group, and the number of patients with dosage adjustment of warfarin after postoperative INR detection was significantly lower compared with the patients in the control group. A study also reported that using genetic testing for *VKORC1* and *CYP2C9* could lead to safer anticoagulation in CF-LVAD patients [6]. These results suggest that genetic testing for warfarin sensitivity possibly has a greater advantage in helping to determine the stable dose of warfarin than the traditional approach during the perioperative period.

In addition, this study compared the incidence of critical INR values, which are closely related to the postoperative bleeding, during the 3 months of postoperative follow-up. Compared with the control group, the incidence of critical INR values was significantly lower (by 3.09-fold) in the experimental group. The incidence/frequency of critical INR values is often used as a surrogate marker for the risk of bleeding complications during warfarin therapy [13]. In our study, we found that 4 patients in control group were died for the bleeding complications and only 1 patient in experimental group was died for the bleeding complications. These data suggesting that rapid stabilization of INR values assisted by genetic testing helps reduce the risk of bleeding complications after AVR.

However, there was no significant difference in the actual incidence of all adverse cardiovascular events at the end of the 3-month follow-up period. This may be related to the relatively small sample size included in this study. Further studies with larger sample size analyzing the influence of the warfarin sensitivity testing on the incidence of this endpoint event are necessary. In addition, currently, genetic testing for warfarin sensitivity is relatively expensive, so it may not be applicable to all patients based on cost effectiveness, and physicians should respect patients’ choice.

In conclusion, preoperative genetic testing of warfarin sensitivity in patients undergoing valve replacement may help to guide the selection of the initial dose of warfarin to ensure a rapid achievement of target INR value and the reduction of the warfarin side effects.

## Data Availability

The data presented in the study are included in the article, further inquiries can be directed to the corresponding author.
